# Anti-Adhesion Activity of A2-type Proanthocyanidins (a Cranberry Major Component) on Uropathogenic *E. coli* and *P. mirabilis* Strains

**DOI:** 10.3390/antibiotics3020143

**Published:** 2014-04-03

**Authors:** Daria Nicolosi, Gianna Tempera, Carlo Genovese, Pio M. Furneri

**Affiliations:** Department of Biomedical Sciences, Section of Microbiology, University of Catania, Via Androne 81, Catania 95124, Italy; E-Mails: dnicolosi@unict.it (D.N.); carlo.genovese@studium.unict.it (C.G.); furneri@unict.it (P.M.F.)

**Keywords:** acute uncomplicated cystitis, cranberry, A2-type proanthocyanidins, *P. mirabilis*, *E. coli*

## Abstract

Urinary tract infections (UTIs) are relatively common in women and may be classified as uncomplicated or complicated, depending upon the urinary tract anatomy and physiology. Acute uncomplicated cystitis (AUC) occurs when urinary pathogens from the bowel or vagina colonize the periurethral mucosa and reach the bladder. The vast majority of episodes in healthy women involving the same bacterial strain that caused the initial infection are thought to be reinfections. About 90% of AUC are caused by uropathogenic *Escherichia coli* (UPEC), but *Proteus mirabilis* also plays an important role. Several studies support the importance of cranberry (*Vaccinium macrocarpon*) proanthocyanidins in preventing adhesion of P-fimbriated UPEC to uroepithelial cells. In this study, we evaluated the *in vitro* anti-adhesion activity of A2-linked proanthocyanidins from cranberry on a UPEC and *Proteus mirabilis* strains and their possible influence on urease activity of the latter. A significant reduction of UPEC adhesion (up to 75%) on the HT1376 cell line was observed *vs.* control. For the strains of *P. mirabilis* there was also a reduction of adhesion (up to 75%) compared to controls, as well as a reduction in motility and urease activity. These results suggest that A2-type cranberry proanthocyanidins could aid in maintaining urinary tract health.

## 1. Introduction

The infections of the urinary tract (UTI) are very common pathologies, both in hospitals and in the community [1]. Community infections are defined as uncomplicated when they are limited to only the lower urinary tract without anomalies; these mainly affect young women due to their anatomy (short urethra with respect to men and easy colonization of the periurethral zone by intestinal bacteria). From 25% to 50% of young women have at least one episode of UTIs in their lives. Of these, about 27% have a relapse during the six months following the first infection, while about 3% have two relapses during the same period [2].

The symptomatology is the same as the first infection: frequent and intense need to urinate, burning and/or pain on urination. The urine is often cloudy, sometimes even pink due to the presence of blood.

Among the risk factors that predispose women to contract recurrent cystitis are frequent sexual relations, having contracted their first UTI at less than 15 years of age and a family history of UTIs. Some studies have also shown that when the first infection is due to *E. coli*, there is a greater probability of having a relapse in the following six months than when the infection is due to other microorganisms [3]. Numerous data suggest that an alteration of the normal vaginal microbiota, in particular when there is a reduction of the lactobacillus population with consequent lowering of the quantity of hydrogen peroxide produced, can be considered a predisposing factor for *E. coli* colonization. Also, for this reason, post-menopausal women can develop recurrent cystitis. The risk factors are the same as those for younger women, with the addition that the lowering of estrogens following menopause causes notable modifications of the vaginal microbiota, in particular a loss of lactobacilli [4]. Recurrent urinary infections have a negative impact on the quality of life of “predisposed” women, both in psychological and economical terms. A woman with recurrent cystitis has to have frequent urine tests, antibiotic treatment and, when prescribed, also preventive therapy.

Preventive antibiotic treatment, which should be started only after having eradicated the pre-existing infection, is carried out, following guidelines, with trimethoprim-sulfamethoxazole, nitrofurantoin, cefaclor or cefalexin, ciprofloxacin or norfloxacin, and fosfomycin. These antibiotics are used at lower doses with respect to those recommended for the treatment of the full-blown infection [5]. Most clinicians recommend continuing therapy for at least six months. Even if these antibiotics are well tolerated over the long-term, the emergence of resistant strains is always a big problem, especially as regards the widely-used broad spectrum antibiotics such as fluoroquinolones [6]. There are, in fact, many on-going studies trying to establish the real contribution of long-term antibiotic therapy to the emergence of multi-resistant strains.

Uropathogenic *Escherichia coli* (UPEC) is the etiological agent in about 90% of community acquired infections and more than 50% of those acquired in hospitals, including those associated with the presence of a catheter. The uropathogenic strains of *E. coli* can be classified in four phylogentic groups called A, B1, B2 and D. Principally, B2 and D cause most extra-intestinal infections, including UTIs. These strains differ from those believed to be harmless commensals of the intestine due to the expression of particular virulence factors that specifically enhance their ability to cause infections of the human urinary tract. Most strains express numerous fimbriae (also called adhesins) diffused on the cell surface, belonging principally to two groups: type 1 fimbriae, mannose-sensitive (MS), which bind to the glycoproteins in the mannose (the virulence factors most frequently expressed by 80% to 100% of the UPEC strains), and P fimbriae, mannose-resistant (MR), which, instead, bind to the α-D-Gal(1,4)-β-D-Gal, a disaccharide of galactose. Adhesins are necessary for the UPEC strains to bind to uroepithelial cells or, eventually, to catheter surfaces. Type 1 fimbriae are those that mediate the initial phases of urinary infection in as much as they use the property of the vesicle mucous, rich in mannose receptors. It is believed that these adhesins are also able to recognize the extra-cellular matrix proteins (collagen, fibronectin, laminin and Tamm-Horsifall proteins) thus distinguishing epithelia from other structures. It also appears that these adhesins can mediate bacterial auto-aggregation and the formation of biofilm that are inducers of the inflammatory response associated with adhesion and to the colonization of UPEC strains.

The P fimbriae, the second most common virulence factor associated with UPEC strains, are associated with the process of invasivity, in as much as they would come into play later to guarantee mannose-independent adhesion, necessary to stop bacteria being eliminated together with the mucous in the urine.

Among the other Gram-negatives there is the species *Proteus*, which are not included among the most common causes of UTIs but play an important role in catheterized patients or those having structural anomalies of the urinary tract. *Proteus* has many virulence factors that include, among others, the notable motility provided by flagella, the production of urease and adhesion to uroepithelial cells mediated by fimbriae [7]. *P. mirabilis* expresses different types of fimbriae, among which are those that are mannose-resistant [8,9]. *Proteus*, instead, is particularly important for its ability to form calcium and magnesium (struvite) stones in the bladder and kidneys following hydrolysis of urea catalyzed by urease [10], and biofilm that is particularly stabile thanks to the action of various fimbriae [11,12].

Numerous studies have recently shown a correlation between the consumption of cranberries (*Vaccinum macrocarpon*) and the possibility of preventing UTIs [13,14,15,16,17]. The mechanism by which cranberries are effective in the prevention and treatment of UTIs has not yet been definitively established, but it seems very probable that it interferes with the mechanisms of bacterial adhesion to uroepithelial cells. If the bacteria are not able to adhere they are not able to colonize the urinary tract. This effect is to be attributed to two components of cranberries: fructose, that blocks Type 1 fimbriae (sensitive to mannose), and the proanthocyanidins (PACs) that, instead, inhibit the P fimbriae (mannose resistant). PACs can be of type A or B, but only those of type A (contained in cranberries) have been associated with anti-adhesive activity against uropathogens [18].

Cranberries are commercially available as: juice, syrup, capsules and lozenges. Their preparation can modify the composition and the contents of PACs [12]. For this reason, in this study we decided to use pure type A2 PACs, in the quantities indicated as effective: 50 µg/mL [19] and at higher and lower dosages ranging from 15 µg/mL to 100 µg/mL.

The aim of our study was to evaluate the inhibiting activity of type A2 proanthocyanidins on strains of *Proteus*, isolated from urine, compared to the already well-known activity on uropathogenic strains of *E. coli.* We also evaluated the effect of type A PACs on the motility of *Proteus* and on the production of urease.

## 2. Results and Discussion

### 2.1. Adhesion Assay

**Figure 1:**
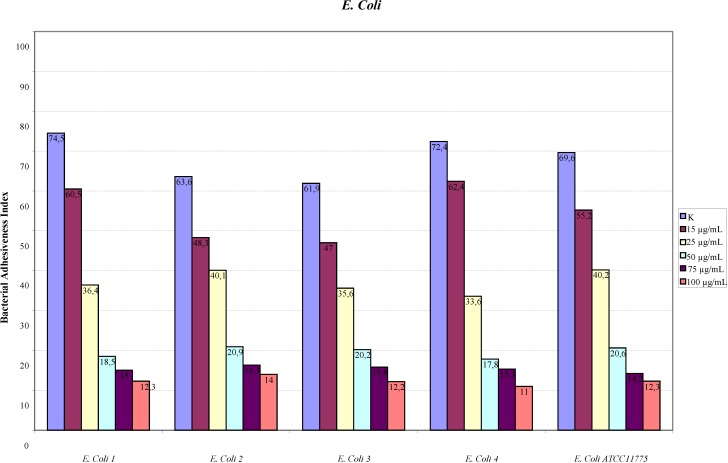
Adhesion indexes for the strains of *E. coli*.

**Figure 2:**
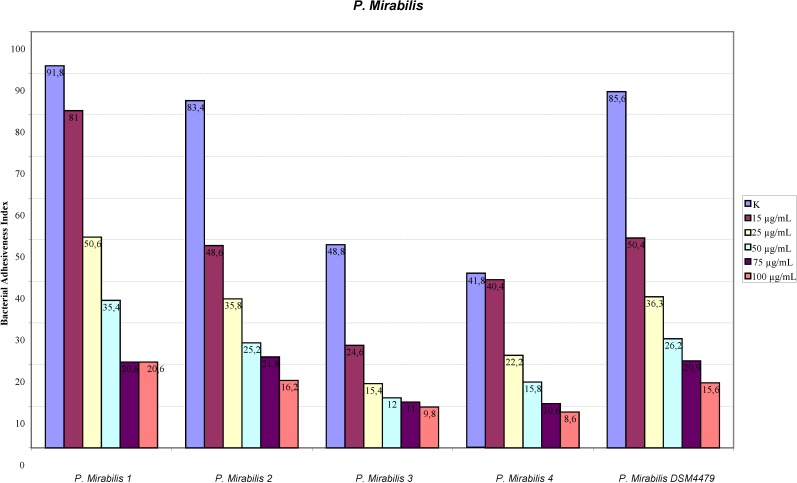
Adhesion indexes for the strains of *P.mirabilis*. In both figures the reduction of the adhesion indexes is clear.

**Figure 3:**
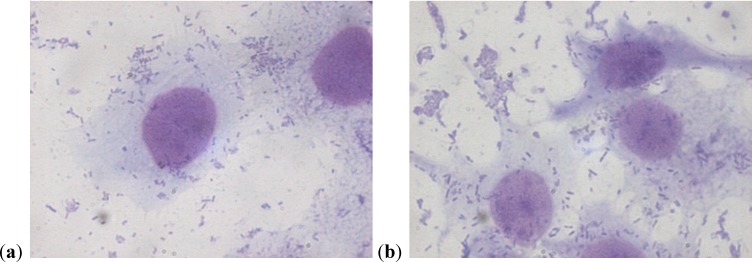
(**a**)(**b**) *P. mirabilis* adherent to a cell on a control slide.

**Figure 4:**
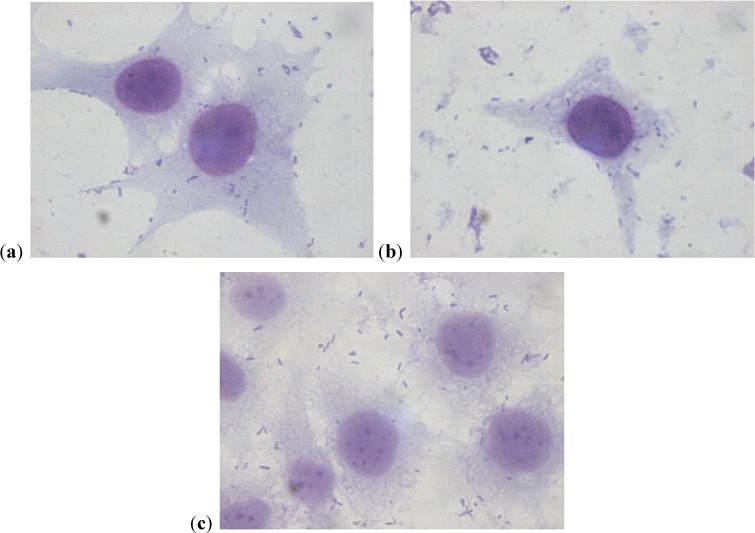
(**a**–**c**) Strains of *P. mirabilis* adherent to cells after treatment with PACs at the concentration of 50 µg/mL. The lower number of adherent bacteria can be clearly seen.

The assay of adhesion carried out on the strains treated with PACs showed a reduction of up to 75% of the adhesion index both for the UPEC strains and for the strains of *P. mirabilis*, with respect to the same strains cultured without PACs. Such a large reduction was obtained only at the concentration of 50 µg/mL. The lower concentrations had a variable efficacy in reducing adhesion of the bacteria in the study, as well as providing discrepant results. At the concentration of 15 µg/mL, *E. coli* shows a decrease between 13.8% and 24.1%, while *P. mirabilis* presents a high variability in its values, with percentages ranging from 3.3% up to 49.6%. With 25 µg/mL, a decrease between 36.9% and 53.6% for *E. coli* and between 44.9% and 68.4% for *P. mirabilis* occurs. With 50 µg/mL, values appear more consistent, with reduction percentages varying from 67.1% to 75.4% for *E. coli* and from 61.4% to 75.4% for *P. mirabilis*. With 75 µg/mL, the observed decrease is between 74.3% to 79.9% for *E. coli* and from 66.7% to 77.4% for per *P. mirabilis*. At the concentration of 100 µg/mL, the reduction goes from 78% up to 84.8% for *E. coli* and from 77.6% up to 81.7% for *P. mirabilis*. These results are shown in Figure 1 and Figure 2 demonstrating the constant decrease of adhesion by all the strains examined. The count of adhering bacteria was carried out manually, both for controls (Figure 3) and treated strains (Figure 4).

### 2.2. Scanning Electron Microscopy

**Figure 5:**
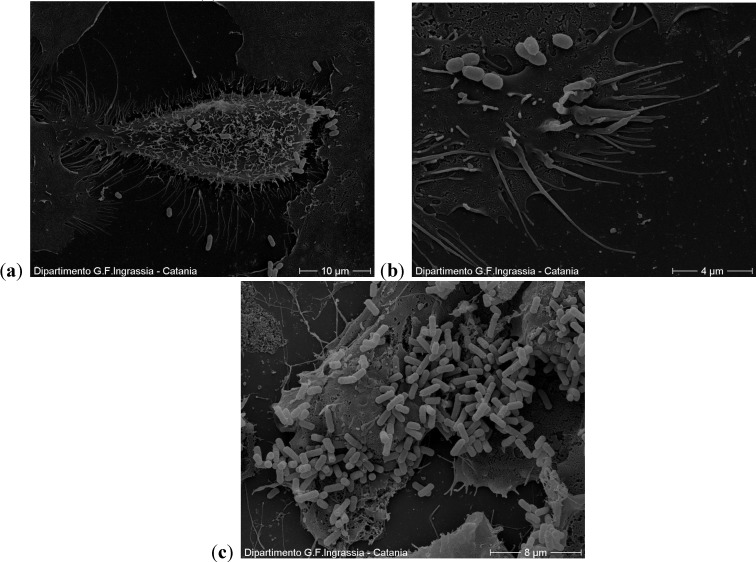
(**a**,**b**) A cell with few adhering bacteria at the concentration of 50 µg/mL. In the outer part, which is in contact with the environment, the cells have cilia, flagella and microvilli; (**c**) In the controls the cells are also integral but with a higher number of adherent bacteria.

From the images obtained from scanning electron microscopy, the cells are integral and the adherent bacteria are fewer than those in the controls (Figure 5).

### 2.3. Motility Test

**Figure 6:**
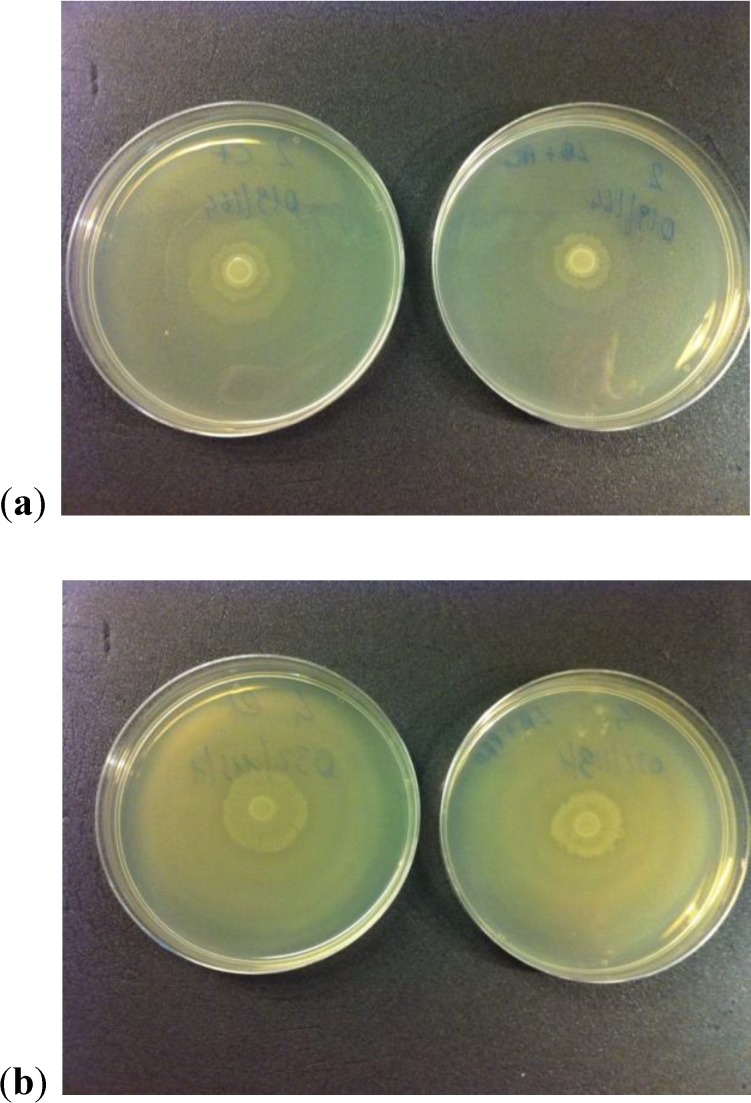
(**a, b**) Reduction of swarming capacity observed for two *P. mirabilis* strains. The lower size of growth halo can be seen in both right plates.

Only for *P. mirabilis* 2 and *P. mirabilis* 4 we observed a reduction of swarming capacity with respect to the control (Figure 6). For the other *P. mirabilis* strains, substantial differences in the swarming were not observed. The cause of this different behavior is unknown at present but will be the subject of further in-depth analysis.

### 2.4. Production of Urease

The capacity to hydrolyze urea, thanks to an enzyme called urease, producing ammonia and carbon dioxide, is considered one of the principal characteristics of the species of *Proteus*. This was thus used as an identifying criterion to distinguish them from other non-fermenting bacteria belonging to the family of the Enterobacteriaceae.

In Christensen’s medium, *Proteus* was able to use the urea as the only source of nitrogen, thus producing a dose of ammonia sufficient to change the color of the medium from yellow to fuchsia. The change of color was due to the presence of red phenol, contained in the medium, which is yellow up to pH 6.8 and then becomes a bright red at pH 8.2.

The absorbance mean value for *P. mirabilis* 3 was 0.075 OD with respect to 0.203 OD of the control. The pH mean value was 7 *vs*. 8.5 of the control. *P. mirabilis* 4 showed the same behavior, with 0.105 as average OD (*versus* 0.417 of the control) and 7.5 as average pH (*versus* 9 of the control) respectively. On the contrary no substantial differences were observed for the remaining two strains examined, neither in absorbance nor in pH. The change of media color, actually, was not evident for both strains, showing a limited urease activity. The causes for the discrepancy in the results observed for the examined strains is still unknown and will be the subject of further analyses.

### 2.5. Discussion

Cranberries have been used for decades for the prevention and treatment of UTIs. Numerous studies have been carried out supporting their efficacy in reducing the number of relapses in women subject to recurrent infections [13,14,15,16,17]. It is well known that their efficacy depends on the anti-adhesive effect of proanthocynidins that they contain on the fimbriae of the uropathogenic strains of *E. coli*. In this study we wanted to demonstrate a similar effect also on the fimbriae expressed by *P. mirabilis*, as well as a reduction of motility and urease activity, two other important virulence factors.

*P. mirabilis* can exist in two different morphotypes: in the first it has 6/10 peritrichous flagella that allow individual movement; in the second it has thousands of flagella that allow a particular type of movement, defined swarming. The two different types of movement alternate during the growth of *Proteus*. When the bacterium is of the second morphotype, the cells move a lot then undergo a temporary halt when the bacterium changes to the first morphotype. The result of this alternation is the characteristic growth in concentric circles that can be seen on the agar dishes. Both these types of movement are made by means of the flagella, the filaments composed principally of a protein called flagellin. The protein FlaA is codified by the *flaA* gene [20] and by *flhD*. It seems that the activity of this gene, over expressed during the phase of swarming, is notably reduced by exposure to PACs [21].

Also, the urease activity is slightly inhibited. By means of hydrolysis of the urea, the bacteria obtains a source of nitrogen but, contemporarily, this leads to the formation of crystals that can block the catheter or damage kidneys and bladder [22,23]. For this reason, infection from *Proteus* causes severe damage to tissues with respect to that from *E. coli*. The activity of cranberries, and of proanthocyanidins in particular, on the species of *Proteus* has to date been poorly investigated [21]. From the results of this study it seems that they could also play an important role in the prevention of infections caused by this microorganism.

## 3. Experimental

### 3.1. Bacterial Strains

For our experiments we used 5 strains of *P. mirabilis* and 5 of *E. coli* isolated from the urine of women with a history of recurrent cystitis, a standard strain of *P. mirabilis* (DSM4479) and a standard strain of *E. coli* (ATCC11775).

### 3.2. Adhesion Assay

The adhesion assay was carried out using the method of Di Martino *et al.* [24] slightly modified. The HT1376 cells, from human bladder carcinoma, were grown on a sterile cover glass in 24-well plates at 37 °C in Minimal Essential Medium (MEM) with fetal bovine serum at 10%, 2 mM of glutamine and 100 µg/mL of streptomycin. Before infection the cells were washed with PBS to remove any antibiotic present in the culture medium.

The bacteria were grown for 36 h at 37 °C in Luria-Bertani broth with the addition of type A proanthocyanidins, at the most efficacious concentration of 50 µg/mL [18] and then at the concentrations of 100, 75, 25 and 15 µg /mL. They were then centrifuged and re-suspended in MEM at a concentration of 0.5 McFarland, which corresponds to 10^8^ CFU/mL. Then the prepared bacterial suspensions were placed in contact with the cells (1 mL of suspension per well) and incubated at 37 °C for 3 h. Finally they were washed three times with PBS to remove any bacteria that had not adhered, the cells were then fixed with methanol, colored with Giemsa at 10% and observed at the microscope. The adhesion index obtained is the average number of adhering bacteria per cell, from an examination of 100 cells. Each test was performed in triplicate.

### 3.3. Scanning Electron Microscopy

Sterile cover glasses similar to those used for adhesion assay were fixed with 3% glutaraldehyde in 0.12 M phosphate buffer solution (pH 7.2) at 4 °C for 1 h. The samples were post-fixed in 1% osmium tetroxide in the same buffer, dehydrated in ethanol, critical point dried and sputter coated with a 5 nm gold layer using an Emscope SM 300. A Hitachi S-4000 field emission scanning electron microscope was used for the observation [25].

### 3.4. Motility Test

To evaluate the activity of type A PACs on the motility of *P. mirabilis*, a drop was removed from the 6 suspensions used for the experiment described above and inoculated in the same number of plates containing Mueller-Hinton agar. As control, a further 6 plates were inoculated with a suspension of bacteria grown in Luria-Bertani broth for 36 h at 37 °C without the addition of PACs. The plates were then incubated for 24 h at 37 °C and the swarming was then observed [26].

### 3.5. Production of Urease

To evaluate the inhibition activity of PACs on the production of urease Christensen’s medium was used in the liquid form, with the addition of a solution of urea at 40%. For the inoculum we used the suspensions already prepared for the adhesion assay in which the 5 wild strains of *P. mirabilis* and the collection strain were grown both in the presence and absence of PACs. A drop of each suspension was inoculated in a test tube containing 9.5 mL of medium and 0.5 mL of urea and incubated for 3 h at 37 °C. Obviously in all the test tubes the change in color from yellow to fuchsia was observed. At this point the suspensions were read at the spectrophotometer at 570 nm to show the difference between the strains used as control and those grown in contact with PACs. The test was repeated three times. Moreover, PH of all solutions was examined through a pH Meter [27].

## 4. Conclusions

This work evaluated the efficacy of type A2 PACs, contained in cranberries, in the prevention of UTIs [28]. Our results show that, other than the well-known activity on uropathogenic strains of *E. coli*, they are active also on strains of *P. mirabilis*, reducing the ability to adhere to the epithelial cells of the bladder. These results indicate that PACs also partly inhibit the activity of the flagella, resulting in reduced motility and urease production. Further studies are needed to better characterize the latter features.
